# Post-incisional ventral hernia repair in patients undergoing chemotherapy: improving outcomes with biological mesh

**DOI:** 10.1186/s12957-016-1011-5

**Published:** 2016-10-06

**Authors:** A. Brescia, F. Tomassini, G. Berardi, M. Pezzatini, A. Dall’Oglio, F. Pindozzi, M. Gasparrini

**Affiliations:** Department of General Surgery, Sant’Andrea University Hospital, Sapienza University of Rome, Rome, Italy

**Keywords:** Incisional hernia, Biological mesh, Chemotherapy, High risk patients, Morbidity

## Abstract

**Background:**

Patients requiring ventral hernia (VH) repair during perioperative chemotherapy have a higher risk for post-operative complications. The aim of the study was to perform a case-controlled analysis in patients undergoing chemotherapy who underwent VH repair using biological mesh or synthetic mesh.

**Methods:**

From January 2013 to December 2015, 32 patients, within 8 weeks from chemotherapy administration, were treated electively for VH repair using a biological mesh (BIOMESH). A control group (CG) receiving chemotherapy within the same time interval and treated with synthetic meshes was selected. There were no differences regarding sex, age, American Society of Anesthesiologists (ASA) score III, BMI, and size of the defect. Morbidity, type of complications, and recurrence rate were investigated and compared between the two groups.

**Results:**

In the BIOMESH group, eight patients (25 %) experienced complications. Wound dehiscence occurred in four (12.5 %) patients and was treated conservatively. Only three small seromas not requiring treatment were observed. The CG presented a higher mean Clavien-Dindo complication grade (1.94 ± 0.44 vs 1.63 ± 0.52; *p* = 0.13) and a higher incidence of wound dehiscence (*n* = 9/32, 28.1 % vs *n* = 4/32, 12.5 %; *p* = 0.11). Five patients developed seroma treated by wound drainage. One patient experienced an intra-abdominal collection treated by percutaneous drainage. At the univariate and multivariate analysis use of traditional mesh, BMI and the ASA III were predictive factors of post-operative complications. Two patients (6.3 %) developed a VH recurrence only in the CG.

**Conclusions:**

Biological meshes could be considered a valid option to improve post-operative short-term outcomes in selected high-risk patients undergoing chemotherapy treated for VH repair.

## Background

Post-incisional ventral hernia (VH) is a common complication after abdominal surgery reaching an incidence that ranges between 2 and 20% according to the literature [1–3]. Several factors are related with its presentation depending on both technical aspects and patient characteristics; type of abdominal incision, suture materials, surgical technique, and type of mesh used to repair the defect are important technical factors to consider as well as advanced age, high BMI, post-operative septic complications and corticosteroid treatment [4, 5].

Patients undergoing oncologic surgery have a higher risk of VH occurrence as a result of several and complex factors related both to the primitive disease and to the surgical procedure itself. Moreover, some of these patients may require VH repair in a short time frame from perioperative chemotherapy due to symptomatic presentation, increasing the risk of post-operative wound infection and recurrence; this is probably related to the immune cells function and thus mesh incorporation that are known to be suppressed under oncological systemic treatments [6, 7]. Indeed, according to the Ventral Hernia Working Group (VHWG) grading system for the assessment of surgical site recurrence risk, every immunosuppressed patient is classified as grade 2 as well as patients with comorbidities [8]. Certainly, the use of synthetic meshes could reduce the incidence of VH recurrence, although the risk of infection limits their use in contaminated surgical fields or in high-risk patients [4, 9].

Biologic materials-based meshes (BIOMESH) have recently emerged as a useful option to repair VH in high-risk patients; these meshes in fact, carry a lower risk of infection and degrade in septic fields thus avoiding the necessity of removal [3, 10]. Piazzese et al. in 2004 demonstrated no differences in terms of wound complications and VH recurrence in liver-transplanted patients undergoing incisional hernia repair comparing synthetic mesh with biological mesh [11]. Moreover, in a recent study, Saied et al. concluded that chemotherapy administration did not increase the rate of wound complications in their series of patients undergoing VH repair with biomaterial mesh [3]. Despite this, no data comparing the use of synthetic mesh with BIOMESH in high-risk patients undergoing chemotherapy are available in the literature.

Due to the lack of data, we decided to perform a case-controlled study concerning high-risk patients undergoing chemotherapy for different malignancies who underwent VH repair with BIOMESH placement; these cases were controlled with patients undergoing VH repair using traditional synthetic mesh. We specifically investigated rate of complications and VH recurrence.

## Methods

From January 2013 to December 2015, 382 patients presented to our department with different post-incisional hernias requiring surgery, and of these, 213 patients (55.7 %) were previously treated for oncological diseases. In the latter, timing and indications to VH repair were reviewed by a multidisciplinary team composed by surgeons, oncologists, radiotherapists, and gastroenterologists considering patient’s characteristics (age and comorbidities) and clinical symptoms.

One hundred and nine patients (51.2 %) receiving different chemotherapy regimens depending on the primitive disease within 8 weeks from VH repair were selected for the study and retrospectively analyzed. This time interval was chosen for the known risk of wound complications after the use of bevacizumab (AVASTIN, Genentech, San Francisco, CA) [6]. All patients presented with symptomatic post-incisional hernias.

Thirty-two patients (31.7 %) were treated with the placement of a porcine derma-derived biological mesh (Fortiva® - Tutogen Medical GmbH, Neunkirchen am Brand, Germany). All patients underwent an open intra-peritoneal underlay technique; the biological mesh was placed beyond the fascial edges with a 5-cm minimum overlap and fixated trans-fascially and circumferentially using separate monofilament absorbable stitches. Fascial defect was then re-approximated or closed when feasible as reinforcement. Two drains were placed subcutaneously and removed after 48 h in all cases.

A control group (CG) of patients receiving chemotherapy within the same time interval, treated with the same technique but using synthetic intra-peritoneal mesh, was created with a 1:1 ratio in order to create a case-control analysis. All patients were defined as American Society of Anesthesiologists (ASA) score II due to their oncological disease. There were no differences between the two groups regarding sex, age, number of ASA score III patients, BMI, and VH size (Table 1). With a median follow-up (FU) of 24 (7–36) months, all adverse events were recorded and classified according to the Clavien-Dindo grade (CD) [12]. Particularly, the presence of wound dehiscence and seroma (collection with no evidence of infection) was classified depending on the necessity of additional use of antibiotics and/or procedures such as post-operative wound opening, percutaneous drainage, or operative debridement. Intra-abdominal collections were defined as abscess whenever fever, leukocytosis, and/or radiological signs of infection were detected.

**Table 1:** Patients’ characteristic*s*

Parameter	BIOMESH group *n* = 32	Control group *n* = 32	*p*
Sex (M/F)	18/14	17/15	1
Pathology (colon/ovarian)	25/7	27/5	1
Bevacizumab (*n*)	3/32	3/32	1
Age (years)	58.63 ± 4.8	59.13 ± 4.4	0.70
Defect size (cm)	7.47 ± 3	7.64 ± 2.5	0.83
BMI (kg/m^2^)	25.4 ± 3.1	25.6 ± 2.3	0.80
ASA score III	46.8 % (15/32)	53.1 % (17/32)	0.77

Incisional hernia recurrence was defined as a bulge or defect at the site of VH repair. Patients with clinical suspect of VH recurrence underwent an abdominal US and/or CT for confirmation.

### Statistical analysis

Continuous variables were presented as mean ± standard deviation or median (range) when appropriate, and categorical variables were presented as frequency (percentage). The one-way analysis of variance (ANOVA) test, the chi-square test, and the Student’s *t* test were used when appropriate for comparisons. Univariate and multivariate analyses were performed through a stepwise logistic regression model using morbidity as a dependent variable. Statistical analysis was carried out using SPSS software (Version 20.0. Armonk, NY: IBM Corp) for MacOsX. The significance level was set at *p* < 0.05.

## Results

Thirty-five out of 64 patients were male (54.7 %), the mean age was of 58.8 ± 4.6 years old and the mean BMI of 25.5 ± 2.8. All patients were defined as ASA score II due to their oncological history, and 50 % of them (*n* = 32/64) were ASA III due to their additional comorbidities such as hypertension in 20 patients (62.5 %), chronic obstructive pulmonary disease (COPD) in eight patients (25 %), and diabetes in six patients (18.7 %).

Fifty-two of the 64 patients (81.3 %) had VH following previous surgery for colorectal cancer while 12 (18.7 %) for ovarian cancer; these were equally distributed among the study groups. Patients’ characteristics are depicted in Table 1.

The most common chemotherapy regimen administered for colorectal cancer was FOLFOX (31/52, 59.6 %); six (11.5 %) received bevacizumab for the presence of liver metastases. All patients with ovarian cancer received Taxol and carboplatin.

Mean post-operative length of stay was 4.1 ± 0.6 days in the BIOMESH group and 4.3 ± 1.1 days in the CG (*p* = 0.6).

In the BIOMESH group, eight patients (25 %) experienced complications with a mean Clavien-Dindo grade of 1.67 ± 0.51 (Table 2). Wound dehiscence occurred in four out of 32 patients (12.5 %) after a mean of 6.7 ± 1.5 days from the operation and were treated conservatively. Resolution was obtained after a mean of 6.6 ± 0.6 days of treatment. Only three patients (9.3 %) experienced a small wound seroma not necessitating treatment.

**Table 2:** Post-operative complications

Type of complications	BIOMESH group *n* = 32	CD grade	Control group *n* = 32	CD grade	*p*
Complications	25 % (8/32)		53.1 % (17/32)		0.02
Wound dehiscence	12.5 % (4/32)	2	28.1 % (9/32)	2	0.11
Seroma	9.3 % (3/32)	1	15.6 % (5/32)	2	0.35
Intra-abdominal collection	0 % (0/32)	–	3.1 % (1/32)	3	–
Subcutaneus hematoma	6.2 % (2/32)	2	9.4 % (3/32)	2	1
Mean Clavien-Dindo grade	1.63 ± 0.52		1.97 ± 0.44		0.13

The CG had a significant higher morbidity rate compared with the BIOMESH group (*n* = 17/32, 53.1 % vs *n* = 8/32 25 %; *p* = 0.02) with a higher, although not statistically significant, mean Clavien-Dindo grade (1.94 ± 0.44 vs 1.63 ± 0.52; *p* = 0.13). The incidence of wound dehiscence was higher in the CG (*n* = 9/32, 28.1% vs n = 4/32, 12.5%; *p* = 0.11) although not statistically significant; the latter were treated conservatively with complete resolution after 6.8 ± 0.7 mean days of treatment. Five patients (15.6 %) developed subcutaneous seroma after 7.5 ± 0.6 mean days from the operation treated by drainage through wound opening. One patient experienced an intra-abdominal collection after 3 days from the operation treated by percutaneous drainage.

At the univariate analysis, the use of synthetic mesh, BMI, age >60 years old, and ASA III were predictive factors of post-operative complications (Table 3). Multivariate analysis was performed using significant predictive factors at the univariate analysis. BMI, use of synthetic mesh, and ASA III were confirmed as predictive factors of post-operative complications.

**Table 3:** Predictive factors of post-operative complications

	Univariate analysis			Multivariate analysis		
Parameter	OR	95 % CI	*p*	OR	95 % CI	*p*
Age >60 years old	1.29	1.11–1.50	0.001	1.04	0.81–1.34	0.73
Male sex	1.27	0.39–4.08	0.68			
BMI	1.71	1.23–2.37	0.001	2.13	1.07-4.23	0.03
Defect size	1.04	0.84–1.29	0.67			
ASA score III	69	7.60–625.83	<0.001	178.71	4.84–632.61	0.005
Synthetic mesh	3.54	1.04–12.06	0.04	48.66	1.157–150.37	0.02

After a median follow-up of 24 months (7–36), no patients in the BIOMESH group developed VH recurrence. In the CG, two patients out of 32 (6.3 %) presented a VH recurrence after a mean of 15 ± 1.4 months from the initial repair. In these patients, the diagnosis of recurrence was clinically suspected and confirmed by a CT scan. These two patients were previously treated with FOLFOX within a mean of 5.5 ± 0.7 weeks from surgery, and only one patient received bevacizumab additionally. No surgical repair of the recurrence was performed in these patients.

## Discussion

Post-operative incisional hernia is the most common long-term complication after abdominal surgery; worldwide consensus concerning optimal timing and surgical strategy is currently lacking; therefore, post-operative VH still represents a challenging issue for surgeons [13, 14]. The use of prosthesis is nowadays considered as a gold standard due to the lower rate of recurrences compared to direct tissue repair. However, currently used meshes such as polypropylene or GORE-TEX can cause adhesions, fistulae, wound infections, mesh contraction and migration, seroma, and chronic pain [9, 15–18]. Furthermore, recurrence rate following VH repair with mesh still represents an issue reaching a rate of 10 % in some series [19]. The incidence of post-operative complications and recurrence is based on a multifactorial etiology: patients’ characteristics, comorbidities (such as COPD and diabetes), and technical aspects (technique, site of mesh placement, and type of mesh used) [4, 5]. In this setting, chemotherapy has been identified as a risk factor for complications and recurrence due to the consequent immunosuppression [6, 7].

The recent diffusion of biologic materials-based meshes has lowered infection rates and overall complication rates in high-risk patients [3, 11]. For these reasons, we performed this retrospective study to evaluate the usefulness and safety of the use of BIOMESH in high-risk patients who underwent chemotherapy in a short time frame.

Considering the two group’s homogeneity regarding important risk factors such as BMI, ASA score III, age, size of the VH defect, and pathology, we observed a statistically significant lower incidence of post-operative complications in the BIOMESH group. These better short-term outcomes are probably related to the intrinsic characteristic of the porcine BIOMESH which provides a collagen and extracellular matrix scaffold in which the host fibroblast enhances angiogenesis and deposits new collagen resulting in a lower risk of infection, erosion, and rejection [9, 20–22]. In patients who underwent chemotherapy, in which the presence of immunosuppression has a negative impact on mesh incorporation, these are crucial aspects to consider. The non-synthetic nature of these prostheses certainly play a role in diminishing the rate of post-operative infections.

After a median follow-up of 24 months (7–36), no patients in the BIOMESH group developed a recurrence while two patients in the CG were clinically and radiologically diagnosed with a recurrence. This result should be strengthened by a study with a bigger sample size and a longer follow-up.

Our univariate analysis concerning the impact of different factors on the development of complications, in addition to confirm age, BMI, defect size, and ASA score III as well-known predictors of morbidity, demonstrated that the use of BIOMESH was protective for these high-risk patients. These results were subsequently confirmed at the multivariate analysis.

The small number of patients certainly represents the main limit of this study. For this reason, the impact of bevacizumab administration on post-operative outcomes was not investigated; only six patients equally distributed between the two groups underwent bevacizumab-associated therapy, and only one developed a recurrence. On the other hand, it has to be considered that patients undergoing VH repair in a short time frame from chemotherapy are highly selected: specifically, it is commonly considered that patients should undergo surgery after an adequate time interval from chemotherapy, in order to reach an appropriate compliance of the patient, improving post-operative results. Despite this, in patients with symptomatic VH, surgical treatment represents the only option. After a careful multidisciplinary evaluation, we enrolled 64 patients, and to our knowledge, this represents the largest case-controlled study available in literature concerning VH repair in these highly selected patients. On the other hand, this could result in a possible selection bias certainly influencing positively our post-operative results due to the selected pool of patients. However, this process could be, in our opinion, hardly avoided due to the particular condition and surgical indication of these patients.

Unfortunately, cost evaluation was not performed in our study due to the lack of data. The retrospective fashion of the study is another limit; certainly, a prospective randomized study could improve and strengthen our results.

## Conclusions

In conclusion, post-incisional ventral hernia repair still represents a challenge for surgeons due to the risk of wound dehiscence and recurrence related to different technical aspects and patient characteristics. The use of biological meshes could be considered a valid option to improve post-operative short-term outcomes in selected high-risk patients undergoing chemotherapy.

## Abbreviations

BIOMESH: Biologic materials-based meshes
CD: Clavien-Dindo grade
CG: Control group
VH: Ventral hernia

## References

[1] Burger JW, Lange JF, Halm JA, Kleinrensink GJ, Jeekel H (2005). Incisional hernia: early complication of abdominal surgery. World J Surg.

[2] Cheng H, Rupprecht F, Jackson D, Berg T, Seelig MH (2007). Decision analysis model of incisional hernia after open abdominal surgery. Hernia.

[3] Saied A, David J, LaBarbera K, Katz SC, Somasundar P (2015). Chemotherapy does not adversely impact outcome following post-incisional hernia repair with biomaterial mesh. J Surg Oncol.

[4] Krpata DM, Blatnik JA, Novitsky YW, Rosen MJ (2013). Evaluation of high-risk, comorbid patients undergoing open ventral hernia repair with synthetic mesh. Surgery.

[5] Le Huu NR, Mege D, Ouaissi M, Sielezneff I, Sastre B (2012). Incidence and prevention of ventral incisional hernia. J Visc Surg.

[6] Gordon CR, Rojavin Y, Patel M, Zins JE, Grana G, Kann B, Simons R, Atabek U (2009). A review on bevacizumab and surgical wound healing: an important warning to all surgeons. Ann Plast Surg.

[7] Rettenmaier MA, Abaid LN, Brown JV, Micha JP, Goldstein BH (2009). Chemotherapy and patient co-morbidity in ventral site hernia development. J Gynecol Oncol.

[8] Breuing K, Butler CE, Ferzoco S, Franz M, Hultman CS, Kilbridge JF, Rosen M, Silverman RP, Vargo D, Ventral Hernia Working G (2010). Incisional ventral hernias: review of the literature and recommendations regarding the grading and technique of repair. Surgery.

[9] Cavallaro A, Lo Menzo E, Di Vita M, Zanghi A, Cavallaro V, Veroux PF, Cappellani A (2010). Use of biological meshes for abdominal wall reconstruction in highly contaminated fields. World J Gastroenterol.

[10] Skipworth JR, Vyas S, Uppal L, Floyd D, Shankar A (2014). Improved outcomes in the management of high-risk incisional hernias utilizing biological mesh and soft-tissue reconstruction: a single center experience. World J Surg.

[11] Piazzese E, Montalti R, Beltempo P, Bertelli R, Puviani L, Pacile V, Nardo B, Cavallari A (2004). Incidence, predisposing factors, and results of surgical treatment of incisional hernia after orthotopic liver transplantation. Transplant Proc.

[12] Dindo D, Demartines N, Clavien PA (2004). Classification of surgical complications: a new proposal with evaluation in a cohort of 6336 patients and results of a survey. Ann Surg.

[13] Butters M, Redecke J, Koninger J (2007). Long-term results of a randomized clinical trial of Shouldice, Lichtenstein and transabdominal preperitoneal hernia repairs. Br J Surg.

[14] Sailes FC, Walls J, Guelig D, Mirzabeigi M, Long WD, Crawford A, Moore JH, Copit SE, Tuma GA, Fox J (2010). Synthetic and biological mesh in component separation: a 10-year single institution review. Ann Plast Surg.

[15] Chrysos E, Athanasakis E, Saridaki Z, Kafetzakis A, Dimitriadou D, Koutsoumpas V, Chalkiadakis G, Xynos E, Zoras O (2000). Surgical repair of incisional ventral hernias: tension-free technique using prosthetic materials (expanded polytetrafluoroethylene Gore-Tex Dual Mesh). Am Surg.

[16] Karakousis CP, Volpe C, Tanski J, Colby ED, Winston J, Driscoll DL (1995). Use of a mesh for musculoaponeurotic defects of the abdominal wall in cancer surgery and the risk of bowel fistulas. J Am Coll Surg.

[17] Leber GE, Garb JL, Alexander AI, Reed WP (1998). Long-term complications associated with prosthetic repair of incisional hernias. Arch Surg.

[18] Voyles CR, Richardson JD, Bland KI, Tobin GR, Flint LM, Polk HC (1981). Emergency abdominal wall reconstruction with polypropylene mesh: short-term benefits versus long-term complications. Ann Surg.

[19] Cassar K, Munro A (2002). Surgical treatment of incisional hernia. Br J Surg.

[20] Hodde J (2002). Naturally occurring scaffolds for soft tissue repair and regeneration. Tissue Eng.

[21] Holton LH, Kim D, Silverman RP, Rodriguez ED, Singh N, Goldberg NH (2005). Human acellular dermal matrix for repair of abdominal wall defects: review of clinical experience and experimental data. J Long Term Eff Med Implants.

[22] Shaikh FM, Giri SK, Durrani S, Waldron D, Grace PA (2007). Experience with porcine acellular dermal collagen implant in one-stage tension-free reconstruction of acute and chronic abdominal wall defects. World J Surg.

